# Anti-Bacterial Adhesion on Abiotic and Biotic Surfaces of the Exopolysaccharide from the Marine Bacillus licheniformis B3-15

**DOI:** 10.3390/md21050313

**Published:** 2023-05-20

**Authors:** Vincenzo Zammuto, Antonio Spanò, Eleonora Agostino, Angela Macrì, Claudia De Pasquale, Guido Ferlazzo, Maria Giovanna Rizzo, Marco Sebastiano Nicolò, Salvatore Guglielmino, Concetta Gugliandolo

**Affiliations:** 1 Department of Chemical, Biological, Pharmaceutical and Environmental Sciences, University of Messina, Viale Ferdinando Stagno D’Alcontres 31, 98166 Messina, Italy; vzammuto@unime.it (V.Z.); eleonora.agostino@studenti.unime.it (E.A.); angela.macri@studenti.unime.it (A.M.); marco.nicolo@unime.it (M.S.N.); salvatore.guglielmino@unime.it (S.G.); concetta.gugliandolo@unime.it (C.G.); 2 Research Centre for Extreme Environments and Extremophiles, Department of Chemical, Biological, Pharmaceutical and Environmental Sciences, University of Messina, Viale Ferdinando Stagno D’Alcontres 31, 98166 Messina, Italy; 3 Laboratory of Immunology and Biotherapy, Department of Human Pathology, University of Messina, Via Consolare Valeria, 1, 98124 Messina, Italy; cdepasquale@unime.it; 4 Department of Experimental Medicine (DIMES), University of Genoa and IRCCS Ospedale Policlinico San Martino, 16132 Genoa, Italy; guido.ferlazzo@unige.it

**Keywords:** antiadhesive, antibiofilm, *Bacillus licheniformis* B3-15, exopolysaccharide, *Pseudomonas aeruginosa*, *Staphylococcus aureus*, Thermophiles

## Abstract

The eradication of bacterial biofilm represents a crucial strategy to prevent a clinical problem associated with microbial persistent infection. In this study we evaluated the ability of the exopolysaccharide (EPS) B3-15, produced by the marine *Bacillus licheniformis* B3-15, to prevent the adhesion and biofilm formation of *Pseudomonas aeruginosa* ATCC 27853 and *Staphylococcus aureus* ATCC 29213 on polystyrene and polyvinyl chloride surfaces. The EPS was added at different times (0, 2, 4 and 8 h), corresponding to the initial, reversible and irreversible attachment, and after the biofilm development (24 or 48 h). The EPS (300 µg/mL) impaired the initial phase, preventing bacterial adhesion even when added after 2 h of incubation, but had no effects on mature biofilms. Without exerting any antibiotic activity, the antibiofilm mechanisms of the EPS were related to the modification of the (i) abiotic surface properties, (ii) cell-surface charges and hydrophobicity, and iii) cell-to-cell aggregation. The addition of EPS downregulated the expression of genes (*lec*A and *pslA* of *P. aeruginosa* and *clf*A of *S. aureus*) involved in the bacterial adhesion. Moreover, the EPS reduced the adhesion of *P. aeruginosa* (five logs-scale) and *S. aureus* (one log) on human nasal epithelial cells. The EPS could represent a promising tool for the prevention of biofilm-related infections.

## 1. Introduction

Bacterial adhesion and biofilm formation on natural and abiotic surfaces have pervasive importance in different fields, such as food spoilage, biofouling, and in human health [1,2,3,4]. Biofilm is an organized community of bacterial cells encased in an extracellular self-produced matrix, mainly composed of exopolysaccharides (EPSs) (40–95%), proteins (1–60%), extracellular DNA (1–10%) and lipids (1–40%) [5,6]. Biofilms confer to bacterial cells less susceptibility to disinfectants and antimicrobial agents and protect them from the host immune response, giving rise to chronic and recurrent infections that are notoriously difficult to eradicate. Biofilm formation is a complex process that begins with the adhesion of bacterial cells to surfaces, which depends on physicochemical interactions, abiotic or biotic surface characteristics, cell surface properties, and hydrophobicity [7,8]. Thus, it is necessary to understand the mechanisms and the physicochemical conditions that control the formation of biofilm in order to reduce the microbiological risk related to its formation. Colonization of surfaces and biofilm formation are usually described by five steps [9]: (i) reversible attachment, planktonic cells are weakly attached to substrate by means weak interactions such as electrostatic, van der Waals or hydrophobic interactions; (ii) irreversible attachment, cells adhere to the substrate and form nascent cell clusters; (iii) biofilm maturation-1, cell clusters mature and become progressively layered, embedded in the auto-produced EPS matrix; this stage is accompanied by the activation of *quorum-sensing* signaling; (iv) biofilm maturation-2, cell clusters reach their maximum thickness, and (v) biofilm dispersion, cells leave the biofilm structure in order to contaminate other surfaces.

The *Pseudomonas aeruginosa* ATCC 27853 and *Staphylococcus aureus* ATCC 29213 have been reported as biofilm models of clinically significant bacteria [10,11,12]. After the initial attachment to a solid support, occurring during the first few hours, bacteria form microcolonies. Thus, a few adhering sessile microorganisms, depending on the substrate surface properties, favor the adhesion of other free-floating bacteria, in the so-called “co-adhesion” phenomenon. Biofilm formation in *P. aeruginosa* depends on a series of coordinated cellular structures and metabolic pathways. Outer membranes lipopolysaccharides and extracellular appendages, such as flagella, type IV pili, and Cup fimbriae, are involved in the initial bacterial attachment to a surface [13]. Moreover, both the bacterial sensing and responding to surfaces after the initial attachment, represent crucial steps in the biofilm formation. The motile-to-sessile transition is often associated with (i) the formation of biofilm, (ii) repressed motility, (iii) irreversible adhesion, and (iv) the production of exopolysaccharides (EPSs) [14]. Reversible adhesion is determined in some strains of *Pseudomonas* by carbohydrate-binding proteins, so-called lectins. In *P. aeruginosa* two types of PA-IL and PA-IIL lectins, synthesized by the *lec*A and *lec*B genes, respectively, were identified [15,16,17]. PA-IIL is positioned in the outer membrane where plays an essential role in the cell-to-cell adhesion, and indirectly in the induction of the *quorum sensing* (QS) response. Activation of QS systems also induced two genes, *pel* and *psl*, that codify for two structurally different EPSs, promoting the irreversible attachment. Pel was described as an N-acetyl glucosamine (GlcNAc)- and N-acetyl galactosamine (GalNAc)-rich polysaccharide [18], whereas Psl, composed of neutral pentasaccharide subunit that contains mannose, rhamnose, and glucose in a 3:1:1 ratio, was reported to play an essential role in preliminary steps of biofilm formation [19,20]. The attachment of *S. aureus* cells to surfaces was reported to be mediated by cell wall-anchored proteins, including the clumping factors ClfA and ClfB [21,22,23]. The expression of the operon *ica* was related to the polysaccharides production involved in the *S*. *aureus* adhesion [24].

The knowledge of the steps involved in the adhesion and biofilm development can help us to understand how to contrast them. The search for biocompounds to prevent the initial bacterial adhesion and the biofilm growth could be essential for human health, as well as for industrial and food-processing activities, in which biofilm formation represents a great concern.

Although involved in the biofilm formation, several bacterial exopolysaccharides, are also able to counteract the adhesion and biofilm formation of a wide spectrum of bacteria [25,26]. Several EPSs produced by thermophilic bacteria, isolated from the shallow hydrothermal vents of Eolian Islands (Italy) were reported to possess interesting physical, chemical and rheological properties, such as thermostability and bioactivities (as antivirals and immunostimulants) and represent attractive products for marine biotechnology and pharmaceutical applications [27,28,29,30,31,32]. Among them, the fucose-rich EPS T14, produced by the marine thermophilic *Bacillus licheniformis* T14 [33], isolated from a vent of Panarea Is., was previously reported as able to strongly inhibit the biofilm formation by multiresistant clinical pathogens [34]. The thermostable, mannose-rich EPS B3-15, produced by the thermotolerant *Bacillus licheniformis* B3-15 isolated from a shallow vent at Vulcano Is. [30,35], was firstly reported as a promising polymer in several biotechnological and pharmacological applications as antiviral and immunomodulatory agent [27,29]. More recently, after optimization of its production, the EPS B3-15 possessed high carbohydrates content (67%), constituted by a disaccharide repeating unit having a manno pyranosidic configuration, and low protein content (5%), mainly attributed to the poly-gamma glutamic acid component [36].

In this study we investigated the effects of EPS B3-15 on the adhesion and biofilm formation of *Pseudomonas aeruginosa* ATCC 27853 and *Staphylococcus aureus* ATCC 29213 on polystyrene and a polyvinyl chloride medical device. Preliminarily, the crude EPS B3-15 was added at different times, corresponding to the different phases of biofilm formation, i.e., initial attachment (T0), reversible attachment (T2) and irreversible attachment (T4 and T8), and after the biofilm development. The EPS antibiofilm mechanisms were evaluated as modification of (i) surface properties, (ii) cell-surface charges and hydrophobicity, and (iii) cell-to-cell aggregation. Moreover, the expression of genes related to the adhesion and biofilm formation of *P. aeruginosa* (*lec*A and *psl*A) and *S. aureus* (*clf*A and *ica*D) and the effects of the EPS on the bacterial adhesion to human nasal epithelial cells were also investigated.

## 2. Results

### 2.1. Antibiofilm Activity of EPS B3-15

#### 2.1.1. EPS Addition on Polystyrene Surfaces at Increasing Concentrations

**Figure 1:**
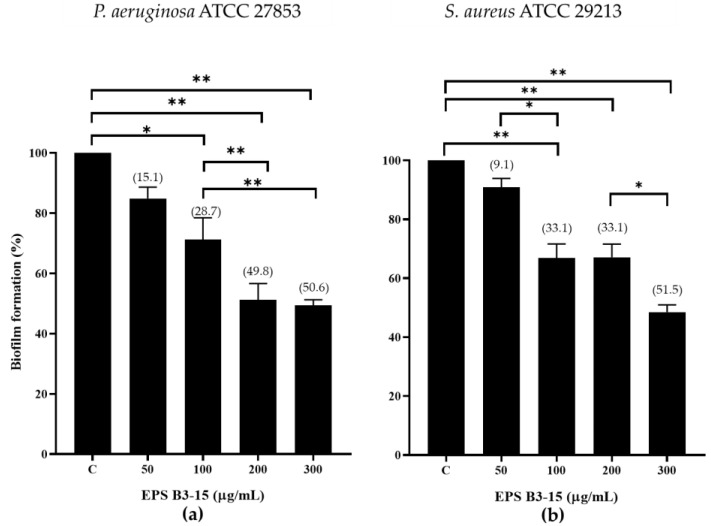
Biofilm formation (%) on polystyrene microplates by (**a**) *Pseudomonas aeruginosa* ATCC 27853 and (**b**) *Staphylococcus aureus* ATCC 29213 in the absence (control, C) or in the presence of EPS B3-15 at increasing concentrations (from 50 to 300 µg/mL). Data represent mean ± SD for six replicates (*n* = 6). Significantly different * *p* ≤ 0.05), ** *p* ≤ 0.01. In brackets are data on biofilm reduction as a percentage.

The inhibition effects of EPS B3-15 at increasing doses (from 50 to 300 µg/mL) on biofilm formation by *P. aeruginosa* and *S. aureus* on polystyrene microplates are reported in Figure 1.

The EPS B3-15 exhibited a dose dependent inhibitory effect on the biofilm formation of *P. aeruginosa* and *S. aureus* (Figure 1), being the concentration of 300 µg/mL the most active in the biofilm reduction of both *P. aeruginosa* (50.6%) and *S. aureus* (51.5%) (Figure 1).

#### 2.1.2. EPS Addition on Polystyrene at Different Times

**Figure 2:**
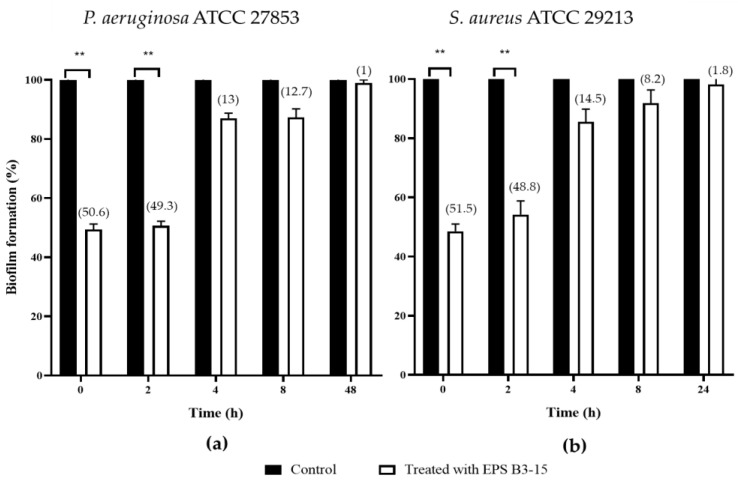
Biofilm formation (%) on polystyrene microplates by (**a**) *Pseudomonas aeruginosa* ATCC 27853 and (**b**) *Staphylococcus aureus* ATCC 29213 in the absence (Control) or after the addition of the crude EPS B3-15 (300 µg/mL) at different times (T0, T2, T4, T8), and after 48 h (T48) for *P. aeruginosa* or 24 h (T24) for *S. aureus*, when the biofilms were completely established. Significantly different ** *p* ≤ 0.01 compared with control (black bar). In brackets are data on biofilm reduction as a percentage.

In order to assess the ability to interfere on the different phases of biofilm formation, EPS B3-15 (300 µg/mL) was added at different times (T0, T2, T4 and T8), and at T48 for *P. aeruginosa* or T24 for *S. aureus*, when their biofilms were completely established (Figure 2).

When added at T0 and T2, EPS B3-15 strongly affected the initial and reversible attachment of *P. aeruginosa* (50.6 and 49.3% inhibition, respectively) and *S. aureus* (51.5 and 48.8% inhibition, respectively).

**Figure 3:**
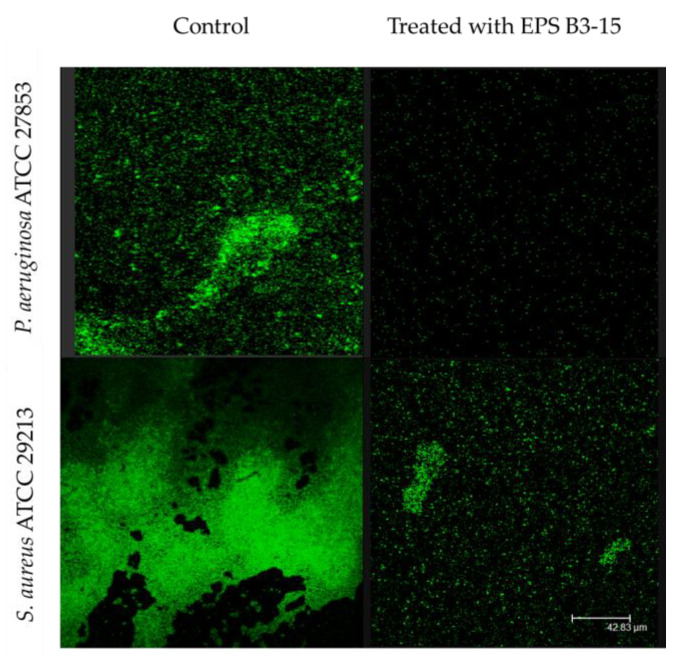
Biofilm formation on polystyrene by *Pseudomonas aeruginosa* ATCC 27853 and *Staphylococcus aureus* ATCC 29213 in the absence (Control) or in the presence of EPS B3-15 (300 µg/mL). Micrographs (×60) after 48 h incubation for *P. aeruginosa* or 24 h for *S. aureus* at 37 °C.

The EPS addition at T4 and T8 exhibited moderate activity during the irreversible adhesion of *P. aeruginosa* (13.0 and 12.7% inhibition, respectively) and *S. aureus* (14.5 and 8.2% of inhibition, respectively). Negligible inhibition was observed on the mature biofilm of *P. aeruginosa* (T48) (1.0%) and *S. aureus* (T24) (1.8%) (Figure 2). After treatment with EPS B3-15, micrographs showed few adherents cells to polystyrene of *P. aeruginosa* and *S. aureus* after 48 and 24 h of incubation, respectively (Figure 3).

#### 2.1.3. EPS Addition on a PVC Medical Device

**Figure 4:**
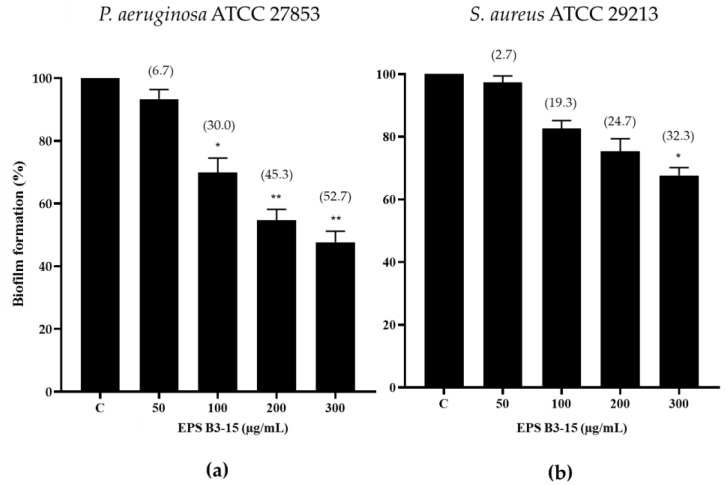
Biofilm formation (%) on a PVC medical device by (**a**) *Pseudomonas aeruginosa* ATCC 27853 and (**b**) *Staphylococcus aureus* ATCC 29213 in the absence (Control, C) and after the addition of EPS B3-15 (from 50 to 300 µg/mL), after 48 or 24 h incubation, respectively. Significantly different * *p* ≤ 0.05, ** *p* ≤ 0.01 compared with untreated controls. In brackets are data on biofilm reduction as a percentage.

The effects of EPS B3-15 (from 50 to 300 µg/mL) addition on the biofilm formation of *P. aeruginosa* and *S. aureus* on a PVC medical device segment are shown in Figure 4.

The EPS at the highest concentration (300 µg/mL) reduced the biofilm formation of *P. aeruginosa* (52.7%) more efficiently than *S. aureus* (32.3%) (Figure 4).

### 2.2. Antibacterial Activity of EPS B3-15 

**Figure 5:**
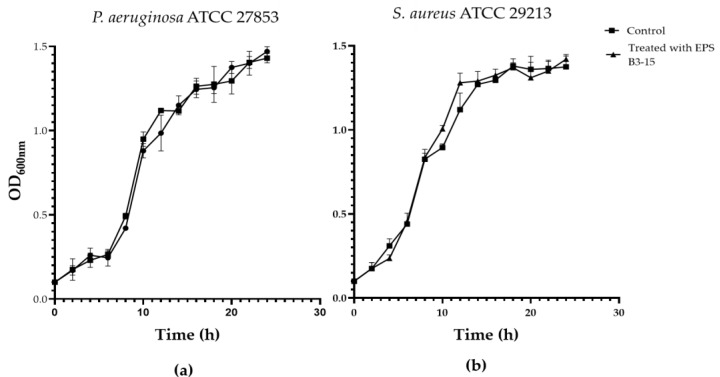
Growth curves of (**a**) *Pseudomonas aeruginosa* ATCC 27853 and (**b**) *Staphylococcus aureus* ATCC 29213 in the absence (Control) and in the presence of EPS B3-15 (300 µg/mL). Data are expressed as averages and standard deviations (*n* = 3).

The EPS (up to 2000 µg/mL) did not affect the growth of *P. aeruginosa* or *S. aureus,* indicating that the biopolymer did not exert any antibacterial activity (Figure S1). The growth curves of *P. aeruginosa* and *S. aureus* evaluated in the absence or in the presence of the EPS (300 µg/mL) are reported in Figure 5.

### 2.3. Effects of EPS B3-15 on Surface Adhesion

#### 2.3.1. Surface Coating Assay

**Figure 6:**
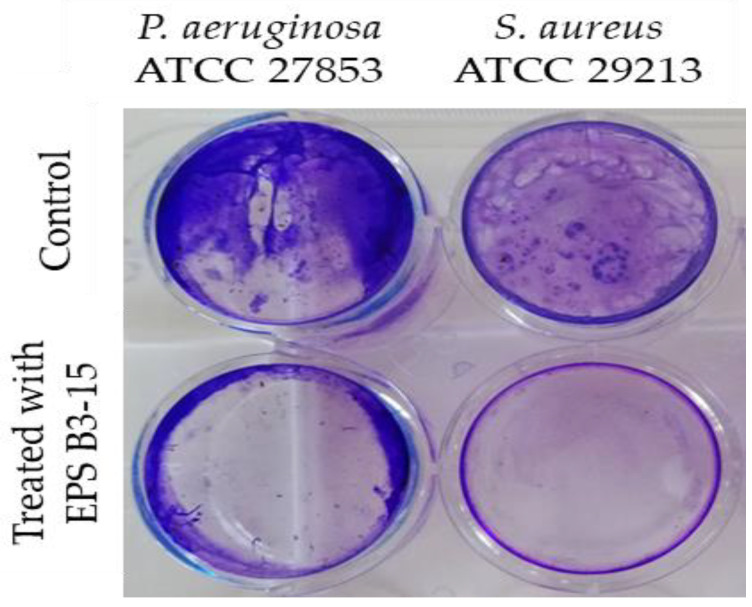
*Pseudomonas aeruginosa* ATCC 27853 and *Staphylococcus aureus* ATCC 29213 adhesions to polystyrene surfaces in the absence (Control) or pre-coated with crude EPS B3-15 (300 µg/mL) after 18 h treatment.

To investigate the effects on abiotic surfaces, the adhesion of *P. aeruginosa* or *S. aureus* was determined on pretreated surfaces with EPS (300 µg/mL). The EPS interfered with the adhesion on polystyrene surfaces of the two strains, and it was effective in preventing the adhesion of *S. aureus* than *P. aeruginosa* (Figure 6).

#### 2.3.2. Cell-Surface Charges and Hydrophobicity Properties

**Figure 7:**
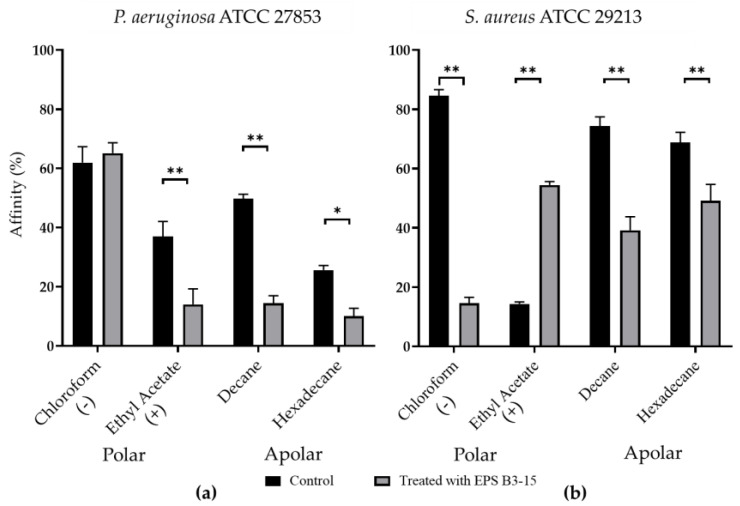
Affinity (expressed as percentage) to polar (chloroform and ethyl acetate) and non-polar (decane and hexadecane) solvents of (**a**) *Pseudomonas aeruginosa* ATCC 27853 and (**b**) *Staphylococcus aureus* ATCC 29213 cells in untreated (Control) or treated with EPS B3-15 (300 µg/mL) conditions. The affinity to chloroform indicates the presence of negative cellular charges (−); the affinity to ethyl acetate indicates the presence of positive cellular charges (+). All assays are representative of three independent experiments. Significantly different * *p* ≤ 0.05, ** *p* ≤ 0.01 compared with untreated controls.

The affinity to polar (ethyl acetate and chloroform) and non-polar solvents (decane and hexadecane) of *P. aeruginosa* and *S. aureus* are shown in Figure 7.

Untreated *P. aeruginosa* possessed high affinity to chloroform (61.9%), low affinity (< 50%) to ethyl acetate, decane, and hexadecane, indicating that cellular surfaces were negatively charged and moderately hydrophobic (Figure 7a). Untreated *S. aureus* cells showed high affinity (84.5%) to chloroform and low (14.2%) to ethyl acetate, whereas the affinity to decane and hexadecane was high (74.4% and 68.3%, respectively), suggesting that cellular surfaces were negatively charged and hydrophobic (Figure 7b).

With the only exception of *P. aeruginosa* to chloroform, EPS B3-15 significantly modified the bacterial cells affinity to the tested hydrocarbons, indicating that the EPS affected both the cell surface charges and the cell wall hydrophobicity.

#### 2.3.3. Cell-to-Cell Aggregation

**Figure 8:**
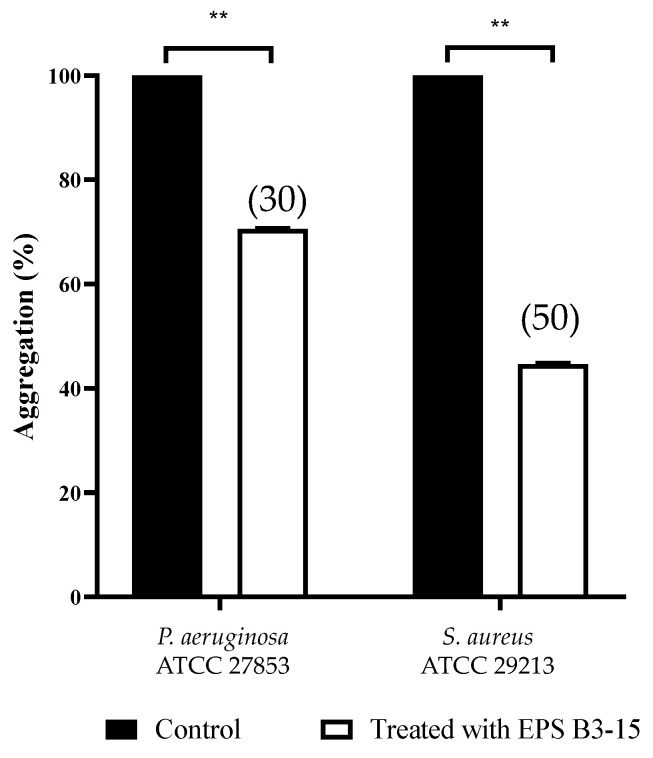
Effects of EPS B3-15 (300 µg/mL) treatment on the cell aggregation (expressed as percentage) of *Pseudomonas aeruginosa* ATCC 27853 and *Staphylococcus aureus* ATCC 29213 after 2 h incubation. Significantly different, ** *p* ≤ 0.01 compared with untreated controls. In brackets are data on aggregation reduction expressed as a percentage.

Cells of *P. aeruginosa* and *S. aureus,* exposed to EPS (300 µg/mL), exhibited less settling (30 and 50%, respectively), due to the inhibition of the intercellular adhesion. Obtained data indicated that EPS inhibits the cell-to-cell aggregation, that is mediated by cell–surface adhesions and therefore it may act as disaggregating agent (Figure 8).

### 2.4. Effects of EPS B3-15 Addition on the Regulation of Adhesion Factors in Biofilm Formation

**Figure 9:**
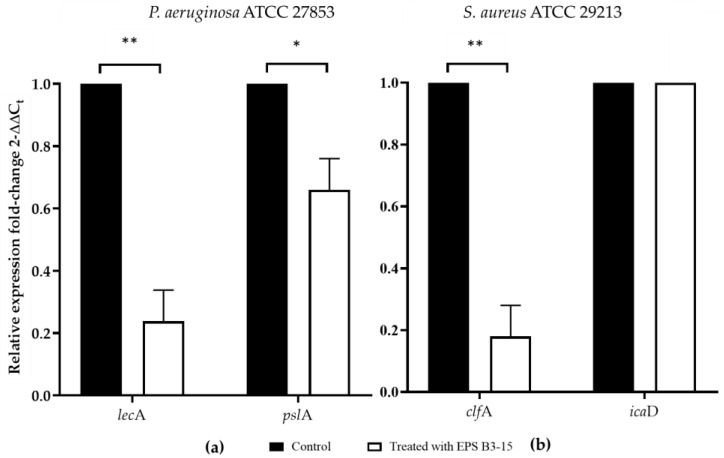
The expression of genes involved in the adhesion and biofilm formation in untreated (Control) or treated with EPS B3-15 (300 µg/mL) by (**a**) *Pseudomonas aeruginosa* ATCC 27853 (*lec*A and *psl*A) and (**b**) *Staphylococcus aureus* ATCC 29213 (*clf*A and *ica*D). Significantly different * *p* ≤ 0.05 or ** *p* ≤ 0.01 compared with untreated controls (Control).

The effects of EPS B3-15 (300 µg/mL) addition on the gene expression of *P. aeruginosa* and *S. aureus* encoding for lectins or adhesins (*lec*A and *clf*A, respectively) involved in the cell-to-cell aggregation and adhesion, and polysaccharides production (*psl*A and *ica*D, respectively), are reported in Figure 9.

After the addition of EPS B3-15, genes *lec*A and *psl*A of *P. aeruginosa* were down-regulated (Figure 9a). Treated with EPS, the *clf*A gene of *S. aureus*, involved in the initial attachment, was downregulated, but *ica*D gene did not (Figure 9b).

### 2.5. EPS B3-15 Addition to Human Nasal Epithelial Cells (HNEpC)

#### 2.5.1. Antiadhesive Effects of EPS Addition to HNEpC

**Figure 10:**
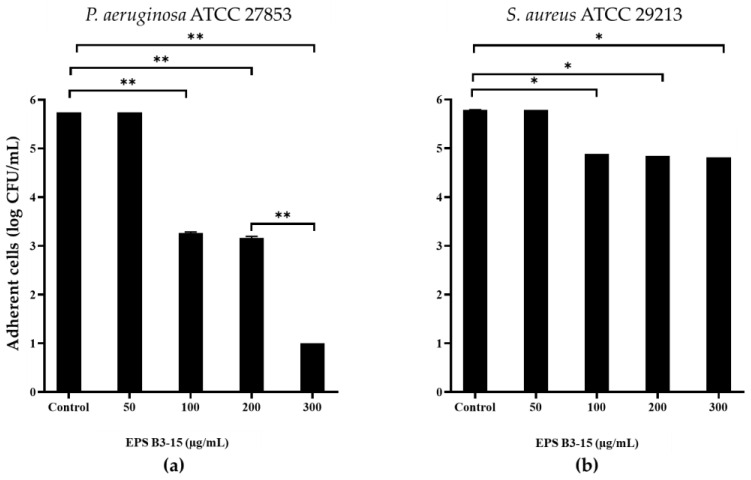
Effects of EPS B3-15 addition at different concentrations on (**a**) *Pseudomonas aeruginosa* ATCC 27853 and (**b**) *Staphylococcus aureus* ATCC 29213 adhesion (expressed as logarithmic scale of CFU/mL) to human nasal epithelial cells, after 2 h incubation at 37 °C. Significantly different * *p* ≤ 0.05, ** *p* ≤ 0.01 compared with untreated controls.

EPS B3-15 addition, at concentrations from 50 to 300 µg/mL, interfered with the adhesion of *P. aeruginosa* and *S. aureus* to HNEpC (Figure 10) after 2 h incubation at 37 °C. At the concentration of 300 µg/mL, EPS reduced the adhesion of *P. aeruginosa* of five log-scale, while the *S. aureus* was reduced of only one log.

#### 2.5.2. EPS Cytotoxicity on HNEpC

**Figure 11:**
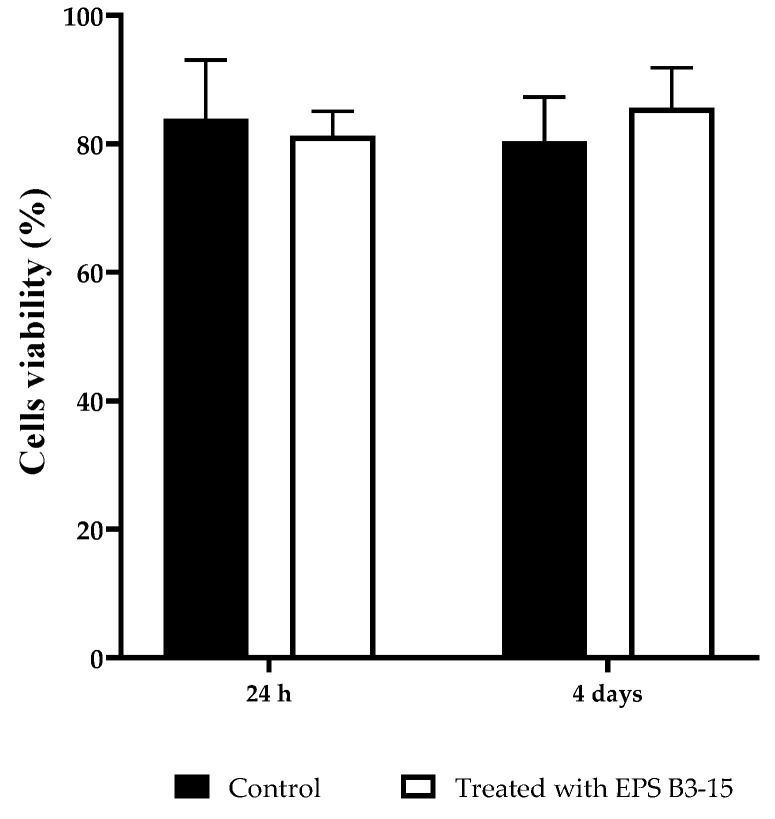
HNEpC viability after 24 h and 4 days in cell culture untreated (Control) or treated with EPS B3-15.

The cytotoxicity of the EPS (300 µg/mL) on HNEpC was evaluated at two incubation times (24 h and 4 days) and it is reported in Figure 11. After incubation with EPS B3-15, the cell viability showed no significative differences with the control cell-culture, indicating noncytotoxic effects also after 4 days.

## 3. Discussion

Bacterial biofilms represent a great concern in a wide range of areas, since most biocides affect free-living bacterial cells, but are scarcely useful against bacteria enclosed in biofilms. Besides their function in the biofilm matrix stabilization and energy storage, bacterial exopolysaccharides are also likely to perform additional functions, such as mediating many of the cell-surface and cell-to-cell interactions that are required for the cohesion and formation of bacterial biofilms [25,37,38]. In this study, we investigated the effects of the mannose-rich EPS B3-15, produced by the thermophilic *Bacillus licheniformis* B3-15, isolated from a hydrothermal vent of Vulcano Is., on the adhesion and biofilm formation of *Pseudomonas aeruginosa* ATCC 27853 and *Staphylococcus aureus* ATCC 29213 on different surfaces (i.e., polystyrene microplates, a polyvinyl-chloride medical device and human epithelial nasal cells), to address its potential use in b iomedical applications. The crude EPS B3-15 was able to impair the adhesion and therefore the biofilm formation on abiotic and biotic surfaces in a dose-dependent manner, without exerting any bacteriostatic or bactericidal activity, similarly to other bacterial antibiofilm polysaccharides identified to date. Other few EPSs from marine strains were reported to possess antibiofilm activity against both Gram-negative and Gram-positive bacteria [34]. The mannose-rich EPS produced by *Oceanobacillus iheyensis* was found active only against the biofilm formation of *S. aureus* (62.3%) on polystyrene but did not against *P. aeruginosa* [39]. The antibiofilm activity of EPS B3-15 was more effective than that of the EPS from the Antarctic sponge-associated strain *Winogradskyella* sp. CAL396, mainly constituted of mannose, that exhibited moderate reduction of the biofilm formation of *P. aeruginosa* (19%) and of *S. aureus* (16%) [11].

The EPS was added at different times (0, 2, 4 and 8 h), corresponding to the initial, reversible, and irreversible attachment, and after the biofilm development. Also when added after 2 h, the EPS (300 µg/mL) impaired the initial phase, strongly reducing (≥50%) the biofilm formation of *P. aeruginosa* and *S. aureus* on polystyrene, but had no effects on mature biofilms. The crucial step of the bacterial cells adhesion to surfaces is influenced by the physicochemical interactions, mainly the Van der Waals forces and the hydrophobic properties of both the abiotic surfaces and bacterial cells. The EPS inhibited the bacterial adhesion on pre-coated polystyrene surfaces, although with some differences for each strain, altering the surface properties (charges and hydrophobicity) and therefore reducing bacteria-surface interactions [40]. Bacterial EPSs present highly variable composition, structures and charged groups on which their specific activities depend. The EPS B3-15 is constituted of disaccharide repeating unit with a manno-pyranosidic configuration and low protein content attributed to the poly-gamma glutamic acid (γ-PGA) component [36]. On the basis of its structure, the EPS could expose negative charges of hydroxyl (carbohydrate) and amidic (γ-PGA) groups that could affect the surface properties, cell surface charges, and lowered cell hydrophobicity, on which bacterial adhesion to surface greatly depends [41,42]. The EPS could act similarly to biosurfactants and bioemulsifiers reported as able to modify the wettability and charge of the surface, and hence affecting the interaction of bacteria with the surface [43,44]. This mechanism of biofilm inhibition is similar to the mode of action of rhamnolipid surfactants produced by *P. aeruginosa* [45], as well as of several biosurfactants and bioemulsifiers produced by marine bacteria displaying antibiofilm activity against pathogenic bacteria [34,46]. Differently on polystyrene surfaces, the EPS reduced the biofilm formation of *P aeruginosa* (52.7%) more efficiently than of *S. aureus* (32.3%) on PVC medical device surfaces. This result could be explained by considering the electrostatic attractions between the surfaces of PVC, which is more negatively charged than the polystyrene [47], and the surface charges of the bacterial cells. Indeed, the cell surfaces of *S. aureus* were more positive after the treatment with EPS, inducing greater adhesion to PVC than *P. aeruginosa*. In addition to the existing preventive procedures, the use of EPS-coated abiotic surfaces, e.g., medical devices, such as orthopedic and endotracheal devices, vascular and urinary catheters, could represent an effective means to counteract bacterial contamination.

The EPS addition reduced cell-to-cell aggregation, mediated by cell-surface adhesins, that is necessary for the first step of bacterial adhesion to surfaces [48], and to epithelial cells and mucosal surfaces [49]. The EPS B3-15 activity on cell-to-cell aggregation was similar to that reported for the purified A101 polysaccharide, produced by the marine *Vibrio* sp. QY101, able to inhibit the intercellular adhesion by both *P. aeruginosa* and *S. aureus* cells [50]. In floating bacterial cells, the EPS addition greatly reduced the expression of *lec*A gene of *P. aeruginosa*, encoding for lectins or adhesins, and of *clf*A gene of *S. aureus,* codifying for surface-anchored protein, confirming that EPS hindered the early steps of the cell adhesion. Furthermore, the EPS partially downregulated the expression of *psl*A, indicating that the polysaccharides biosynthesis, required for the irreversible attachment of *P. aeruginosa* to a variety of surfaces [51,52], was reduced. Conversely, the EPS did not interfere with the expression of *ica*D gene of *S. aureus*, suggesting that the polysaccharides production involved in development of the biofilm formation was not reduced. Future, extensive transcriptomic analyses could be carried out to elucidate better the genetic responses of *P. aeruginosa* and *S. aureus* to the EPS treatment. These results could explain the different mechanisms of EPS action on the biofilm formation by *P. aeruginosa* and *S. aureus* also on biotic surfaces, where the adhesion is also receptor mediated (i.e., adhesins-tissutal matrix). The EPS inhibited the adhesion of *P. aeruginosa* (five logs) more efficiently than *S. aureus* (one log) to the human nasal cells (HNEpC), representing the first target of bacterial infections air-mediated [53]. These results suggest that the addition of the EPS, as a nasal spray, could prevent both the biofilm formation and the dissemination of dispersed cells from mature biofilm on mucous surfaces.

According to the evidences so far suggested, two main hypothetical antibiofilm modes of action of the nonbiocidal EPS B3-15, may be proposed: (i) it acts on surface properties, cell-surface charges and hydrophobicity, (ii) it may act as disaggregating agent, inhibiting the cell-to-cell aggregation, as confirmed by the downregulation of the gene expression of *P. aeruginosa* and *S. aureus* encoding for lectins or adhesins (*lec*A and *clf*A, respectively) involved in the cell-to cell aggregation and adhesion. The mechanism of action of EPS B3-15 appears to be independent from *quorum sensing*, since its presence did not interfere with the luminescence of *Vibrio harveyi* strain G5 [36].

Together with the absence of cytotoxicity in HNEpC, these data suggest that this EPS could be addressed for different biomedical applications, even at high temperatures. EPS B3-15 was recently reported as able to mediate the green synthesis of silver and gold nanoparticles with antimicrobial properties towards Gram-positive and Gram-negative bacteria (*Staphylococcus aureus*, *Escherichia coli* and *Pseudomonas aeruginosa*), as well as fungi (i.e., *Candida albicans*) [54]. In future perspectives, this EPS could be successfully utilized combining its antiviral and immunomodulatory properties [27,32] with the here described antiadhesive ability, in nanotechnological and material science applications (i.e., functionalized devices and nasal spray), as novel strategies to counteract infections.

## 4. Materials and Methods

### 4.1. Bacillus licheniformis Strain B3-15 and EPS B3-15 Production 

*Bacillus licheniformis* strain B3-15, as producer of EPS B3-15, has been previously described [30]. Briefly, the strain was isolated from a fluid sample emitted at 0.7 m depth from a shallow hydrothermal vent located at the Porto di Levante of Vulcano Is. (Italy). B3-15 grew aerobically from 25 to 60 °C and its optimal temperature occurred at 45 °C. The pH range for growth was 5.5–9, with the optimum at pH 7, moreover, the strain grew in a range 0–7% (*w/v*) of NaCl and optimally with 2% (*w/v*) NaCl. The partial 16S rRNA gene sequence was submitted to GenBank under accession number: KC485000. The strain is routinely grown on Tryptic Soy Agar (TSA, Sigma Aldrich, Milan, Italy) plates, plus 1% NaCl (1.5% NaCl final concentration) and frozen at −80 °C in 40% (*v/v*) glycerol for long-term storage.

The EPS B3-15 was produced in flask containing the novel medium SG17, with 5% of glucose as carbon source, incubated at 45 °C for 48 h under shaking conditions at 250 rpm, as previously reported [36]. The yield of the EPS was 240 mg/L. Briefly, the culture was centrifuged at 8000× rpm for 10 min, and the cell-free supernatant (CFS) was obtained by filtering through a 0.2-µm-pore-size membrane (Biogenerica, Catania, Italy). To inactivate enzymes responsible for EPS degradation, the CFS was heated at 100 °C for 20 min. To precipitate the EPS, the CFS was treated with an equal volume of cold absolute ethanol added dropwise under stirring in ice bath, held at −20 °C overnight, and then centrifuged at 10,000× rpm for 30 min. After washing two times with ethanol, it was dissolved in hot water (80–90 °C) and dialyzed (6.00–8.00 KDa-cutoff membrane SpectraPor^(R)^ Standard Grade RC Membrane), lyophilized and weighed. Carbohydrate content was evaluated by phenol–sulfuric acid method, using glucose as standard, and total protein content was estimated using the Bradford reagent (Bio-Rad) and bovine serum albumin as standard [29]. The crude EPS possessed high carbohydrate content (67%), constituted by a disaccharide repeating units having a manno-pyranosidic configuration, and low protein content (5%), mainly attributed to the poly-gamma glutamic acid component [36].

### 4.2. Bacterial Pathogens

*Pseudomonas aeruginosa* ATCC 27853 and *Staphylococcus aureus* ATCC 29213 strains were purchased from the American Type Culture Collection (LGC Promochem, Teddington, United Kingdom). *P. aeruginosa* was routinely maintained into Luria Bertani broth (LB, Sigma Aldrich) and LB solified with 2% Bacto agar (Difco, Baltimore, MD, USA). *S. aureus* was maintained in Tryptic Soy Broth or Tryptic Soy Agar (TSB, TSA). Strains were kept frozen at −80 °C in 40% (*v/v*) glycerol for long-term storage.

### 4.3. Antibiofilm Activity of EPS B3-15

#### 4.3.1. EPS Addition on Polystyrene at Increasing Concentrations

The antibiofilm activity of EPS B3-15 against *P. aeruginosa* and *S. aureus* was evaluated in 96-well polystyrene microplates (Falcon^®^, Fisher Scientific, Milan, Italy), as previously reported by O’Toole et al. [55]. Overnight cultures (180 μL) of *P. aeruginosa* grown in LB or *S. aureus* grown in TSB were poured into microwells (six replicates, OD_600nm_ = 0.1 equivalent to 4.5 × 10^7^ CFU/mL, as experimentally determined) and EPS B3-15 (20 μL) dissolved in Phosphate Buffer Saline (PBS, Sigma Aldrich) at different final concentrations (50, 100, 200 or 300 µg/mL), or 20 μL of PBS as control, were added in each well.

#### 4.3.2. EPS Addition on Polystyrene at Different Times

To evaluate the ability to interfere on the biofilm formation of *P. aeruginosa* and *S. aureus*, the EPS B3-15 at the concentration with the highest antibiofilm activity, was added at different times (0, 2, 4 and 8 h), and after 48 h for *P. aeruginosa* or 24 h for *S. aureus*, when the biofilm was completely established.

Microplates were incubated at 37 °C for 48 h (for *P. aeruginosa*) or 24 h (for *S. aureus*) without shaking. Nonadherent bacteria were removed by washing five times with distilled water. Biofilms were stained with 0.1% (*w*/*v*) crystal violet solution for 20 min. Excess stain was removed by aspiration, and then the plates were washed (5 times) and air-dried (for 45 min). The stained biofilms were solubilized with absolute ethanol and the biofilm mass was spectrophotometrically determined (OD_585nm_) by the level of the crystal violet present in the de-staining solution, using a microtiter plate reader (Multiskan GO, Thermo Scientific, Waltham, MA, USA).

The reduction of biofilm formation of each strain was expressed as antibiofilm activity (%) by applying the following formula:

(1) $$\mathrm{Reduction}\;\mathrm{of}\;\mathrm{biofilm}\;\mathrm{formation}\;\left(\%\right)=\frac{{\mathrm{OD}}_{585\mathrm{nm}\;\mathrm{control}}-{\;\mathrm{OD}}_{585\mathrm{nm}\;\mathrm{sample}}}{{\mathrm{OD}}_{585\mathrm{nm}\;\mathrm{control}}}\times 100$$

Each data point was averaged from six replicated microwells, and the standard deviation (SD) was calculated. Statistical significance (** *p* ≤ 0.01 or * *p* ≤ 0.05) was determined by one-way ANOVA.

To directly observe the multicellular structures into the biofilms onto polystyrene surface, with or without the addition of EPS, the confocal Laser Scanning Microscopy, using a TCS SP2 microscope (Leica Microsystems Heidemberg, Mannheim, Germany), equipped with Ar/Kr laser, and coupled to a microscope (Leica DMIRB) was used. Aliquots (180 µL) of overnight cultures of *P. aeruginosa* in LB or *S. aureus* in TSB (adjusted to OD_600nm_ = 0.1) were distributed into each well containing sterile polystyrene strip (0.5 cm × 1 cm) of a 96-well microplate (Falcon no. 353047). After the addition of the EPS (20 μL) dissolved in PBS (300 μg/mL final concentration), the microplates were incubated at 37 °C for 24 h (for *S. aureus*) or 48 h (for *P. aeruginosa*). Not-attached bacteria were removed by washing with PBS, and the adherent cells on the polystyrene strips were stained with 20 µg/mL of each SYTO9 and propidium iodide (LIVE/DEAD Bac-light Thermo Fisher Scientific, Waltham, MA, USA). After incubation at 30 °C for 5 min in the dark, the strips were microscopically observed.

#### 4.3.3. EPS Addition on a PVC Medical Device 

To evaluate the biofilm formation on PVC medical device without or in presence of EPS B3-15 at different concentration (50, 100, 200 or 300 µg/mL), the crystal violet assay was performed. Sterile segments (1 cm × 0.5 cm) of the PVC tube (external and internal diameters 4.1 and 3 mm, respectively) (BENIFIS SRL, Genova, Italy) were placed in each well of a 96 well microplate, containing 180 µL of each culture (OD_600nm_ = 0.1) and 20 µL of PBS, as control, or EPS B3-15 at final solution of each different concentration were added. The microplates were incubated at 37 °C for 48 h (for *P. aeruginosa*) or 24 h (for *S. aureus*). Each PVC segment was washed with PBS, to remove the non-adherent bacterial cells, stained with 0.1% (*v/v*) crystal violet solution, and washed (5 times) with deionized water. After drying stained biofilms were solubilized with ethanol for 30 min at room temperature, and the de-stained solution was spectrophotometrically measured (OD_585nm_), and the reduction of biofilm formation was calculated as reported above.

### 4.4. Antibacterial Activity of EPS

Minimum inhibitory concentration (MIC) values were determined in microplates using the serial dilution assay, as accepted by the European Committee for Antimicrobial Susceptibility Testing (EUCAST) of the European Society of Clinical Microbiology and Infectious Diseases [56]. Serial dilutions of the EPS (2, 1, 0.5, 0.25 and 0.125 mg/mL) were prepared in MHB, and then inoculated with suitable aliquots of an overnight culture (OD_600nm_ = 0.1) of each strain in MHB, the microplates were incubated at 37 °C overnight and then the growth was estimated spectrophotometrically (OD_600nm_). To confirm the inhibition activity of the EPS, 100 µL from wells without visible growth, were inoculated onto LB or TSA plates for *P. aeruginosa* and *S. aureus*, respectively, and incubated overnight at 37 °C.

The effects of EPS B3-15 on the bacterial growth were spectrophotometrically determined. Aliquots (180 µL) (OD_600nm_ = 0.1) from each overnight strain culture in Muller Hinton Broth (MHB, Sigma Aldrich) were distributed in 96-well polystyrene microtiter plates (six replicates), and 20 μL of EPS B3-15 (300 μg/mL final concentration, in PBS) or PBS used as control, were added to each well. The microplates were incubated at 37 °C for 24 h without shaking and OD_600nm_ values were registered each 2 h.

### 4.5. Effect of EPS B3-15 on Surface Adhesion 

#### 4.5.1. Surface Coating Assay

To evaluate the effects on the polystyrene surface, a volume of 20 μL of EPS B3-15 diluted in PBS (300 μg/mL final concentration), or 20 μL of PBS used as control, were deposited into the center of each well of a 24-well polystyrene microtiter plate (Falcon no. 353047) [57]. The plates were incubated for 30 min at 37 °C to allow the complete evaporation of the liquid. Each well was filled with 1 mL of diluted overnight bacterial culture (containing 10^5^ CFU/mL) in LB for *P. aeruginosa* or TSB for *S. aureus*. After incubation at 37 °C for 18 h in static conditions, the wells were emptied gently, washed with distilled water, and stained with 1 mL of 0.1% crystal violet solution. Stained biofilms were washed with distilled water and air dried to remove the excess of crystal violet, and finally photographed.

#### 4.5.2. Cell-Surface Charges and Hydrophobicity Properties

Cell-surface properties of *P. aeruginosa* and *S. aureus,* based on the bacterial adhesion to hydrocarbons (MATH), were evaluated according to Bellon-Fontaine et al. [58]. Cells from overnight bacterial cultures (grown in LB or TSB, respectively) at 37 °C, were harvested by centrifugation (6000× rpm for 10 min) and washed twice with PBS. Each bacterial suspension (2 mL) was EPS B3-15 (300 μg/mL in PBS) treated for 30 min or with PBS as control. Treated and untreated bacterial suspensions were centrifuged (6000× rpm for 10 min) and resuspended in PBS (OD_400nm_ = 0.5–0.7) (A0). Aliquots of 3 mL of each treated and untreated bacterial suspension were added to each tube containing 0.4 mL of the following hydrophobic solvents (Sigma Aldrich): ethyl acetate, a strong basic solvent; chloroform, an acidic solvent which exhibits negligible basic character; decane, as a non-polar solvent having intermolecular attraction comparable to that of ethyl acetate, and hexadecane, as a non-polar solvent having intermolecular attraction comparable to that of chloroform. After vigorous agitation by vortex, phases were allowed to separate for 10 min at 30 °C, and the OD_400nm_ of the aqueous phase was measured (A1). All assays are representative of three independent experiments. 

The percentage of affinity to hydrocarbons was calculated as follows:

(2) $$\%\;Affinity=\left(\frac{A0-A1}{A0}\right)\times 100\;$$

#### 4.5.3. Cell-to-Cell Aggregation 

The cell-to-cell interactions were performed according to Malik et al. [59] with some modifications. Cells from overnight *P. aeruginosa* and *S. aureus* cultures were harvested by centrifugation (8000× rpm for 10 min) and resuspended in 3 mL of PBS (OD_600nm_ = 0.6). The EPS (300 μg/mL final concentration) or PBS as control, were added to each bacterial suspension, and then tubes were incubated at 37 °C for 2 h, without agitation. Aliquots of 1 mL from each bacterial suspension were spectrophotometrically measured (OD_600nm_). The percentage of aggregation was calculated as follows:

(3) $$\mathrm{Aggregation}\left(\%\right)=1-\frac{{\mathrm{OD}}_{600\mathrm{nm}}{\;}_{\mathrm{FINAL}}}{{\mathrm{OD}}_{600\mathrm{nm}}{\;}_{\mathrm{INITIAL}}}\times 100$$

### 4.6. Effects of EPS B3-15 Addition on the Regulation of Adhesion Factors in Biofilm Formation

#### 4.6.1. Gene Expression Analysis 

The global regulatory pathways of the biofilm formation have been investigated using https://www.genome.jp/kegg/pathway.html (17 January 2023). Genes related to adhesion and biofilm formation of *P. aeruginosa* and *S. aureus* were selected and reported in Table 1. Specificity, the temperature of melting (Tm) and thermodynamic characteristics of each primer were performed using the Multiple Primer Analyzer–Thermo Fisher Scientific and confirmed by Vector NTI program (version 10.3.0; Thermo Fisher Scientific).

The optimization of the PCR conditions was validated for DNA of *P. aeruginosa* ATCC 27853 and *S. aureus* ATCC 29213 and *rpo*D gene (RNA polymerase sigma factor RpoD) has been used as a housekeeping gene for relative quantitation [62,63].

#### 4.6.2. RNA Isolation and Reverse Transcription

*P. aeruginosa* and *S. aureus* were grown in 15 mL polystyrene tubes (Falcon) containing 5 mL of LB or TSB medium, incubated at 37 °C for 48 or 24 h respectively, in static conditions, in the presence of EPS B3-15 (300 µg/mL) or PBS as control. To obtain the RNA, 2 mL of each culture was centrifuged (8000 rpm × 10 min) and the bacterial cells were suspended in Trizol Reagent (Life Technologies, Carlsbad, CA, USA), according to the manufacturer’s instructions. Residual DNA was removed by digestion for 30 min at 37 °C with 1 U of RNase-free DNase (Promega Corporation, Madison, WI, USA). The reaction was stopped by adding 1 μL of RQ1 DNase stop solution and the tubes were incubated at 65 °C for 10 min. RNA samples were quantitatively analyzed using a Nanodrop Spectrophotometer (Thermo Fisher Scientific, Waltham, MA, USA). A total of 300 ng of RNA was used for complementary DNA (cDNA) synthesis. The reverse transcription (RT) was carried out in a 20 µL reaction mixture, containing 1 × reaction buffer, 0.5 mM dNTP, 20 pmol primers, 3 mM MgCl_2_, 20 U RNase inhibitor, and 200 U Improm II reverse transcriptase (Promega Corporation, USA). The reaction was performed using an initial incubation at 25 °C for 5 min, followed by incubation at 37 °C for 60 min and finally at 70 °C for 15 min. After reverse transcription, the cDNA of each sample was directly analyzed in Real-Time PCR.

#### 4.6.3. Relative Quantization of Genes Expression (qRT-PCR)

Relative quantization of genes expression was evaluated using quantitative Real-Time Polymerase Chain Reaction (qRT-PCR). qRT-PCR was carried out in a 20 µL reaction mixture containing 3 µL of cDNA preparation, 0.5 µM of each forward and reverse primers and 10 µL of SsoAdvanced universal SYBR1 Green supermix (2×) (Bio-Rad Laboratories, Hercules, CA, USA). The amplification was carried out using a 7500 Fast Real-Time PCR System at the following conditions: 3 min at 95 °C, followed by 40 cycles of 15 s at 95 °C, 45 s at the temperatures reported in Table 1 for each primer, and finally 40 s at 60 °C. A melting curve analysis was performed using the instrument default settings.

All samples were tested in triplicate and relative expression levels were assessed using the 2^−ΔΔCt^ (Ct) method. The results were expressed as fold change (increase or decrease) of gene expression in the bacterial culture treated with EPS compared to the transcripts in not treated cultures. 

### 4.7. Effects of EPS B3-15 Addition to Human Nasal Epithelial Cells (HNEpC)

#### 4.7.1. Human Nasal Epithelial Cells 

The confluent monolayer of Human Nasal Epithelial Cells (HNEpC, PromoCell, Cat. Num. C-12620, Heidelberg, Germany) was used for all experiments. The human cells were grown in RPMI 1640 (Invitrogen Cergy Pontoise, France) supplemented with antibiotics (penicillin and streptomycin) and 10% fetal calf serum, in 75 cm^2^ flask and incubated at 37 °C with 5% CO_2_ incubator. Confluent cell monolayers were treated with 5 mL trypsin EDTA, resuspended at concentration of 2.5 × 10^5^ cells/mL in culture medium and inoculated in each well of 96 well cell culture plate. Cells were incubated until confluent monolayers were obtained.

#### 4.7.2. EPS Effects on Bacterial Adhesion to HNEpC

The adhesion of *P. aeruginosa* and *S. aureus* to HNEpC was evaluated according to Fernandes de Oliveira [64]. Briefly, the RPMI was removed from cells confluent monolayers in 96-well, and HNEpC were washed three times with RPMI 1640 without antibiotics and serum (RPMI-1). Aliquots of 100 µL of *P. aeruginosa* or *S. aureus* suspensions in RPMI-1 (1.0 × 10^6^ CFU/mL) were added to HNEpC. To evaluate the effect of EPS on the bacterial initial attachment (T0), in each well the EPS, dissolved in RPMI-1 at different final concentrations (50, 100, 200 and 300 µg/mL), or RPMI-1 used as control, was added to HNEpC and the microplates were incubated for 2 h at 37 °C with 5% of CO_2_. To remove non-adherent bacterial cells, the plate was washed two times with RPMI-1 and then with PBS. To lyse HNEpC monolayer cells, 100 µL of cold distilled sterile water were added, and 100 µL containing lysed cells and adherent bacterial cells were recovered, immediately ten-fold serially diluted in PBS, and then plated onto cetrimide agar (Difco, BD Life Sciences) for *P. aeruginosa* or onto mannitol salt agar (Oxoid) for *S. aureus*. The plates were incubated at 37 °C for 18–24 h to determine the CFU/mL of adherent bacterial cells to HNEpC previously treated or not treated with the EPS.

#### 4.7.3. EPS Cytotoxicity

To evaluate the cytotoxicity of the EPS, the viability of HNEpC treated or not treated with the EPS (final concentration of 300 μg/mL) were incubate for 24 h and 4 days, at 37 °C with 5% CO_2_ incubator. After that, the cells were stained with a diluted solution (1:2000, *v*/*v*) of TO-PRO3 (Thermo Fisher Scientifics) and incubated at 4 °C for 15 min in the dark. Viability of stained cells was assessed by using flow cytometry (FACS Canto II).

## 5. Conclusions

Thermophilic bacteria inhabiting Eolian shallow hydrothermal vents have been proven to produce novel exopolymers with unique properties, such as structural complexity, biocompatibility and biological properties, attractive for marine biotechnologies and pharmaceutical applications [32,36,43].

At very low concentration, EPS B3-15 (300 µg/mL) affected the adhesion of *Pseudomonas aeruginosa* and *Staphylococcus aureus* to polystyrene, a PVC medical device and human nasal epithelial cells at the early stage of biofilm formation. Without exerting any antibiotic activity or interfering with *quorum sensing* [36], the EPS acted in different ways: (i) modifying the abiotic surfaces and altering the physical properties of Gram-negative and Gram-positive bacterial cells surfaces, (ii) inhibiting the cell-to-cell aggregation, and (iii) downregulating the expression of genes *lec*A and *psl*A of *P. aeruginosa*, and *clf*A gene of *S. aureus.* Together with the physicochemical modifications, the expression pattern could justify the inhibition of the early bacterial adhesion on polystyrene but did not the preformed biofilm. 

As antibiofilm agent, the EPS inhibited the adhesion of *P. aeruginosa* and *S. aureus* on human nasal epithelial cells, that makes it suitable for the prevention of biofilm-related infections in human diseases. Due to its potential biological roles, also including antiviral and immunomodulatory effects, and antiadhesive properties, the EPS B3-15 may have numerous applications in industry and medicine purposes, as nasal spray or EPS-coating surfaces for medical devices (i.e., orthopedic and endotracheal devices, vascular and urinary catheters).

## Figures and Tables

**Table 1:** PCR target genes and primer sequences.

	Target Gene	Primer Sequences	PCR Annealing Temperature	Amplicon Size (bp)	Reference
*P. aeruginosa* ATCC 27853	*lec*A-PA-I galactophilic lectin	F 5′-GAAGCAGGGCAGGTAACGTC-3′	58 °C	277	This work
		R 5′-CGGGCACGTCGTTGTAGATA-3′			
	*psl*A-biofilm formation protein	F 5′-ACACGGGCTGGATTCATCG-3′	56 °C	245	This work
		R 5′-CAGGCGAAGAACATGATGCG-3′			
*S. aureus* ATCC 29213	*clf*A-clumping factor A	F 5′-ATTGGCGTGGCTTCAGTGCT-3′	56 °C	288	[60]
		R 5′-CGTTTCTTCCGTAGTTGCATTTG-3′			
	*ica*D-intercellular adhesion D	F 5′- ATGGTCAAGCCCAGACAGAG-3′	56 °C	198	[61]
		R 5′AGTATTTTCAATGTTTAAAGCAA-3′			

## Data Availability

Not applicable.

## Supplementary Materials

The following supporting information can be downloaded at: https://www.mdpi.com/article/10.3390/md21050313/s1, Figure S1. Effects of the EPS B3-15 addition at increasing concentrations (from 125 to 2000 µg/mL) on the growth (OD_600nm_) of (a) *P. aeruginosa* and (b) *S. aureus*. Data are expressed as averages and standard deviations (*n* = 3).
