# Supplementary material for: Anti-Bacterial Adhesion on Abiotic and Biotic Surfaces of the Exopolysaccharide from the Marine Bacillus licheniformis B3-15
Source: Mar Drugs. 2023 May 20;21(5):313. doi: 10.3390/md21050313 (PMC10223316; doi:10.3390/md21050313)
Supplement: Supplementary file 1 [file marinedrugs-21-00313-s001.zip › marinedrugs-2340409-supplementary.pdf]

Anti-bacterial adhesion on abiotic and biotic surfaces of the exopolysaccharide from the marine *Bacillus licheniformis* B3-15

Vincenzo Zammuto [1,2], Antonio Spanò [1,2,*], Eleonora Agostino [1], Angela Macrì [1], Claudia De Pasquale [3], Guido Ferlazzo [4], Maria Giovanna Rizzo [1,2,*], Marco Sebastiano Nicolò [1,2], Salvatore Guglielmino [1,2] and Concetta Gugliandolo [1,2]

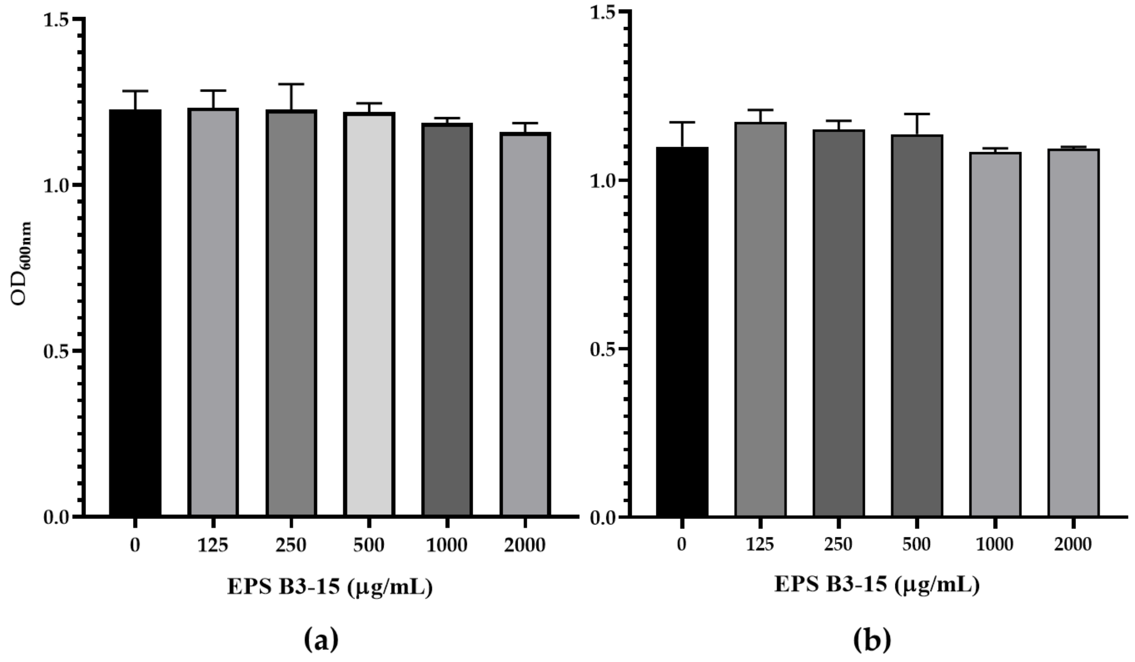

Figure S1. Effects of the EPS B3-15 addition at increasing concentrations (from 125 to 2000 µg/mL) on the growth (OD_{600nm}) of *P. aeruginosa* (a) and *S. aureus* (b). Data are expressed as averages and standard deviations (n= 3).
